# Supplementary material for: Psychometric properties of the Post Traumatic Stress Disorder Checklist for DSM-5 (PCL-5) in Greek women after cesarean section
Source: PLoS One. 2021 Aug 13;16(8):e0255689. doi: 10.1371/journal.pone.0255689 (PMC8363016; doi:10.1371/journal.pone.0255689)
Supplement: Attachment — Submitted filename: Replies to Reviewers comments.docx [file pone.0255689.s002.docx]

**Replies to Reviewers comments**

**Dear Reviewer #1:**

- The Introduction section is too long. I think that the detailed description of PTSD touching all criteria are unnecessary and should be focused on those details that are appropriate to the results. The historical presentation of PTSD is absolutely an unnecessary surplus in the MS. PTSD is relatively common entity in perinatal context, but the description of the PTSD symptoms, questionnaires should be more concise.

We shortened the Introduction part. The sentence *’So far there are no studies assessing the psychometric properties of the PCL-5, in its Greek translation, applied on a special group of postpartum women’’* was moved from the end of the third paragraph to the end of the ‘Introduction’. Thank you for your suggestions.

In the Materials and Methods section:

- 'Medical dossier' is not a good expression. Medical charts are the proper word.

The word has been replaced

In the Ethics sub-section: the second meaning is unnecessary ('After ...')

The second meaning deleted

- If the authors carried out test-retest reliability, then they should present the results.

Unfortunately, there were no test-retest reliability analyses.

- Were there any back and forth translation and pilot testing of the questionnaire to test the comprehension?

Yes, we did back and forth translation, but not pilot testing of the questionnaire.

- Description of LEC-5, DSM-5 and PCL-5 can be shortened.

We did it, with an additional small correction of PCL-5, added from a deleted part of the ‘Introduction’: *“A provisional PTSD diagnosis can be made if a score of each item is 2 or above (range 0 to 4), when there is as score of one or more in the categories of criteria B and C and two or more in categories D and E, and by summing the score (range 0-80) for each of the 20 items.”*

- In Data Analyses: 'Descriptive statistics ...' sentence is unnecessary.

It is deleted.

- The Discussion is a little short and may complete with PCL-5 Factor analysis results in other populations and the consequence of the factor structure.
- We extended the Discussion, according to your note.

**Dear Reviewer #2:**

- I think it need to mention about the limitations of this study more. For example, the author states that "Reported levels of PTSD among participants were low, since the data were positively skewed" in the results, but the effect of this on the results has not been considered. In addition, the participants were two types by Caesarean Sections received, and the impact of this on results should be stated.

We added new information to the ‘Conclusions’, according to your note. Thank you.

**Dear Reviewer #3:** 
Major concerns:
1)Please describe the translation process of the evaluation scale in more detail. Please show the back translation version of PCL-5 and consider the difference from the original version of one. Moreover, please consider the Linguistically limitations of the Greek version of PCL-5. Also, if there are any points that you paid particular attention to during the translation process, please describe them. In addition, please add the Greek version of PCL-5 as an attachment file (an image file is also acceptable).

Translation process is additionally described. Thank you for directing us to this. I also attach the Greek version of PCL-5

2)The strength of this study is the large number of samples. On the other hand, too many samples are statistically more likely to cause type I errors (prone to false statistically significant differences). Please estimate the sample size required for this study from prior probabilities and consider whether the number of samples is appropriate.

‘’Suggested minimums for sample size include from 3 to 20 times the number of variablesand absolute ranges from 100 to over1,000.’’ -*Daniel J. Mundfrom, Dale G. Shaw & Tian Lu Ke (2005) Minimum Sample Size Recommendations for Conducting Factor Analyses, International Journal of Testing, 5:2, 159-168, DOI: 10.1207/s15327574ijt0502_4.* This suggests that our sample size of 469 participants for performing factor analyses is excellent.

Minor concern:

1) Please describe a brief review of the psychosocial characteristics of the Greek postpartum women after Cesarean Section, comparing them to other countries.

So far in Greece the only research that has been carry out on postpartum disorders in women after cesarean section is our survey, published on March 2020 in MDPI: “*Orovou E, Dagla M, Iatrakis G, Lykeridou A, Tzavara C, Antoniou E. Correlation between Kind of Cesarean Section and Posttraumatic Stress Disorder in Greek Women. Int J Environ Res Public Health [Internet]. 2020 Jan [cited 2020 Mar 6];17(5):1592. Available from:* [*https://www.mdpi.com/1660-4601/17/5/1592*](https://www.mdpi.com/1660-4601/17/5/1592)*”*
